# Quantifying the primary and secondary effects of antimicrobial resistance on surgery patients: Methods and data sources for empirical estimation in England

**DOI:** 10.3389/fpubh.2022.803943

**Published:** 2022-08-08

**Authors:** Nichola R. Naylor, Stephanie Evans, Koen B. Pouwels, Rachael Troughton, Theresa Lamagni, Berit Muller-Pebody, Gwenan M. Knight, Rifat Atun, Julie V. Robotham

**Affiliations:** ^1^The National Institute for Health Research (NIHR) Health Protection Research Unit in Healthcare Associated Infection and Antimicrobial Resistance at Imperial College London, London, United Kingdom; ^2^Department of Infectious Disease Epidemiology, Faculty of Epidemiology and Population Health, Antimicrobial Resistance (AMR) Centre, London School of Hygiene and Tropical Medicine, London, United Kingdom; ^3^Healthcare Associated Infection, Fungal, Antimicrobial Resistance, Antimicrobial Usage and Sepsis division, United Kingdom Health Security Agency, London, United Kingdom; ^4^Nuffield Department of Population Health, Health Economics Research Centre, University of Oxford, Oxford, United Kingdom; ^5^The National Institute for Health Research Health Protection Research Unit in Healthcare Associated Infections and Antimicrobial Resistance at the University of Oxford, Oxford, United Kingdom; ^6^Department of Global Health and Population, Harvard TH Chan School of Public Health, Harvard University, Boston, MA, United States; ^7^Department of Global Health and Social Medicine, Harvard Medical School, Harvard University, Boston, MA, United States

**Keywords:** antimicrobial resistance, secondary effects, surgical site infection, surgery, burden

## Abstract

Antimicrobial resistance (AMR) may negatively impact surgery patients through reducing the efficacy of treatment of surgical site infections, also known as the “primary effects” of AMR. Previous estimates of the burden of AMR have largely ignored the potential “secondary effects,” such as changes in surgical care pathways due to AMR, such as different infection prevention procedures or reduced access to surgical procedures altogether, with literature providing limited quantifications of this potential burden. Former conceptual models and approaches for quantifying such impacts are available, though they are often high-level and difficult to utilize in practice. We therefore expand on this earlier work to incorporate heterogeneity in antimicrobial usage, AMR, and causative organisms, providing a detailed decision-tree-Markov-hybrid conceptual model to estimate the burden of AMR on surgery patients. We collate available data sources in England and describe how routinely collected data could be used to parameterise such a model, providing a useful repository of data systems for future health economic evaluations. The wealth of national-level data available for England provides a case study in describing how current surveillance and administrative data capture systems could be used in the estimation of transition probability and cost parameters. However, it is recommended that such data are utilized in combination with expert opinion (for scope and scenario definitions) to robustly estimate both the primary and secondary effects of AMR over time. Though we focus on England, this discussion is useful in other settings with established and/or developing infectious diseases surveillance systems that feed into AMR National Action Plans.

## Introduction

Surgical site infections (SSIs) place a substantial burden on healthcare systems (1). SSIs, known to be “infections of superficial or deeper tissue occurring within 30 days of non-implant surgery, or within 1-year for implant-related procedures” (2), are costly to the National Health Service (NHS) in England, creating prolonged hospital stays for patients and increased costs for hospitals (3). When considering the additional costs of lost productivity and reduced workforce, the economic burden could be substantially more.

Antimicrobials have allowed us to develop safe patient-care pathways that would previously have put patients at a high risk of SSIs, by the integration of protocols of antibiotic prophylaxis into these pathways. Consequently, the threat of antimicrobial resistance (AMR) goes beyond a reduction of treatment effectiveness for acute infections. Increased AMR reduces the effectiveness, and therefore benefits, of the antimicrobial prophylaxis currently protecting patients from potential infections. For example, increasing the risk of SSIs for those undergoing surgery (4, 5). AMR then has the potential to disrupt the care of these patients through multiple processes, often referred to as the “secondary effects” of AMR (4, 6). Incorporating all such potential future costs for different evaluations of associated interventions is key for efficient policy making. To quantify the burden of these secondary effects of AMR from healthcare system and societal perspectives, potential patient health outcomes, payer and provider cost, and socio-economic data are needed (4).

Primary use of health and economic data can be defined as “use for intended clinical, public health, societal and/or research purposes stated *a priori,”* and secondary for purposes other than those stated as primary (7). The primary and secondary use of such data has been highlighted as a key way to tackle AMR through increased knowledge of epidemiology and economic burden, subsequently helping shape the allocation of finite resources. For example, the Global Antimicrobial Resistance Surveillance System was launched in 2015 to promote the collecting of not just microbiological but also outcome data associated with AMR (8), whilst the Wellcome Data Re-use Prize promoted the secondary use of similar AMR data in generating policy recommendations (9).

Though there have been discussions on the importance and use of SSI surveillance data in terms of preventing and improving quality of care in relation to SSIs and surgery patients correspondingly (10), the use of such data in informing the quantification of the secondary effects of AMR has not yet been discussed in detail. There have been reviews that include the quantification of AMR burden in relation to SSIs (11, 12). However, these focus on narrow definitions of AMR burden in relation to SSIs, namely the direct effectiveness of AMR in preventing and treating infections in past or current cohorts of surgical patients. As we have seen from the recent COVID-19 pandemic, there are potentially major costs borne through a capacity or risk threshold being met. There may be an implicit or explicit “occupancy of hospital beds” or “risk of death” threshold being met in unmitigated AMR scenarios. As such, broader treatment behaviors for many patients, and the general population, may need to be changed. For instance, canceling elective procedures in hospitals could in turn can lead to other health and economic burdens to society (13).

We aim to provide a practical discussion of approaches and data sources that have been and/or could be used to estimate the total primary and secondary effects of AMR in relation to surgery, based on literature and available data. In England there is a wealth of national-level data on SSIs, AMR, hospital admissions, population demographics and economic measures (2, 14–16), and as such, this will be the setting utilized. The objectives of this study are to; (i) discuss previous methods used to estimate the primary and secondary effects of AMR in relation to surgery, (ii) discuss potential health and economic data sources available in England, and (iii) based on the methods and data described, propose a conceptual model for quantifying the potential total burden of AMR on surgery patients in England.

## Potential health and economic data in england for quantifying total secondary effects

**Figure 1:**
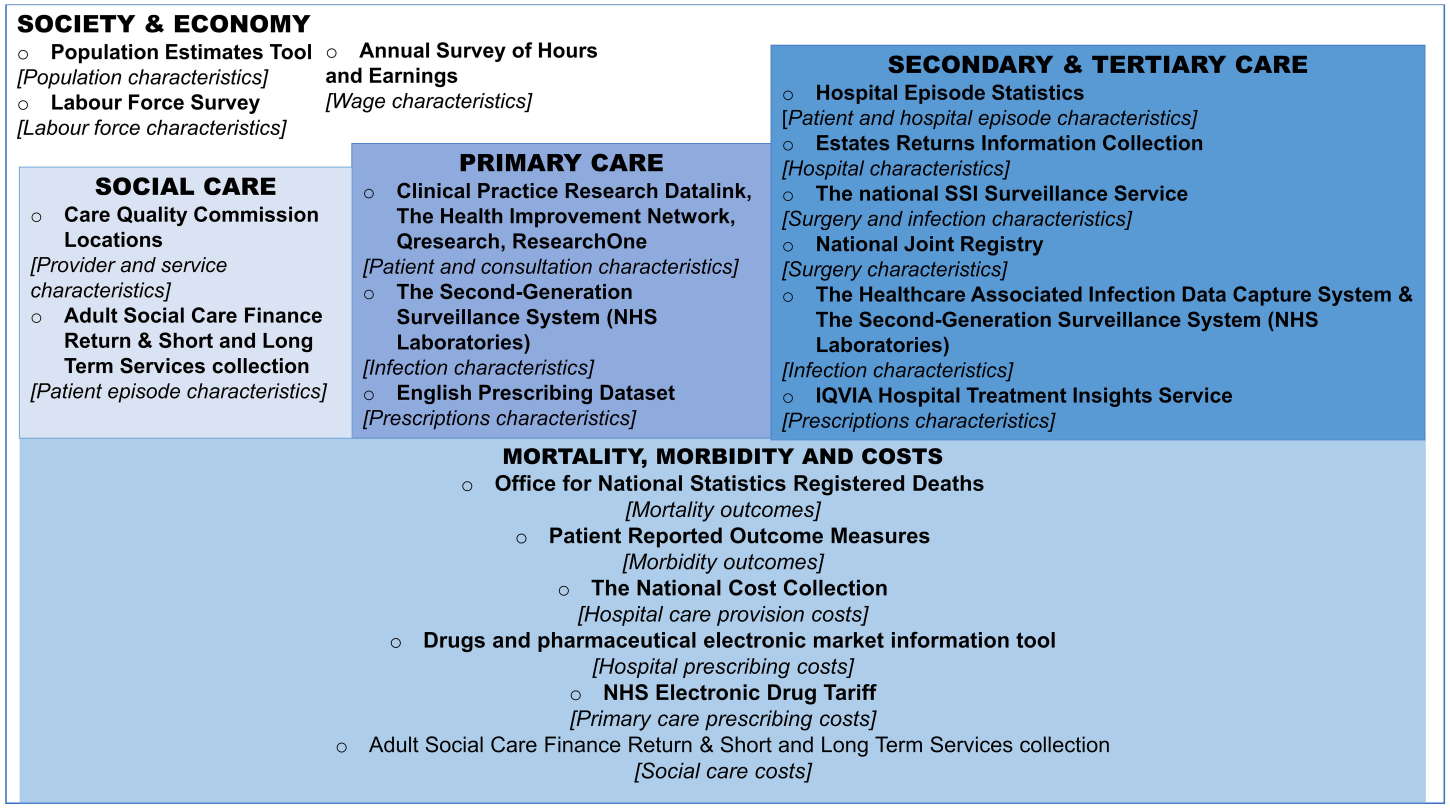
Overview of datasets for the estimation of antimicrobial resistance impact on surgery patients in England. Boxes represent the setting and/or type of the data, bullet points represent the name of the dataset, followed by a high-level description the use of the dataset in square brackets, for the purposes of Surgical Site Infection and Antimicrobial Resistance research. For more information on data sources (see Supplementary Table A1). NHS, national health service; SSI, surgical site infection.

Figure 1 (expanded in Supplementary Table A1) summarizes some of the key datasets available for epidemiology and health economics research for AMR and SSIs in England, highlighting the large breadth of data sources across the healthcare system and wider economy.

In regards to health data, England has a centralized administrative data capture and processing system for hospital admissions and care, known as Hospital Episode Statistics (HES) (16). Secondary use of HES data has, in the past, included the linkage of HES data to other datasets such as to those listed in Figure 1 under “Primary Care” and “Mortality, morbidity and costs.” This can be done to have a more complete picture of patients and their care. For example, as HES collects only data based on what happens within hospitals, further information might be needed on post-discharge mortality. Therefore, HES has been linked with Office for National Statistics (ONS) mortality data to incorporate post-discharge mortality (17). Additionally, HES data can be linked to Second Generation Surveillance System (SGSS) data to get more information about infection characteristics (such as microbe type and susceptibility to antibiotics), whilst antibiotic prescribing data and hospital characteristics may be available through linkage with related datasets (see Supplementary Table A1). This is possible due to granularity of these data capture systems, namely the inclusion of patient-level identifiers (such as unique NHS numbers, names, and date of birth) and hospital identifiers (unique provider codes). A patient-level data set, linked across these sources, therefore could be used to estimate the transition probabilities of patients acquiring types of infections, undergoing revision surgery, and the subsequent impact of these different treatment pathways on mortality, as done previously (18).

A previous review suggests the use of prospective, matched cohort studies to estimate the burden of SSIs by infection type (12). However, such studies are resource intensive and can have low external validity unless conducted on a national/international scale. Secondary use of these national microbiology and HES data sources paired with appropriate statistical methods have been utilized in the past to estimate associated mortality from healthcare-associated infections (19–21), and this could be further expanded to capture post-discharge mortality rates (17).

Linkage of hospital patient data to primary care data (see Figure 1) could allow for exploration on the need for additional patient pathways, such as increased primary care identification of infections, treatment and/or visits following certain infections or procedures. However, although post-discharge Surveillance is encouraged through the national SSI Surveillance Service (SSISS) (2), there can be a delay from the initial procedure to the time that the associated infection is detected, making it harder to define case exposures. For example, there could be up to 1 year from surgery to infection for surgeries requiring the placement of an implant, e.g., artificial joints. When such patients do require hospitalization there is no guarantee that patients will return to the same healthcare facility in which they underwent the related surgery, so any records of infections and surgical procedures need to be cross-referenceable within and between healthcare facilities. This means long-term surveillance of SSIs is required. If basing such estimates on established primary care administrative systems, all three of the primary-care-based administrative systems rely on voluntary inclusion from GP practices and patients, with varying degrees of sample sizes and representativeness across the three systems (22). However, these have still been used for previous analyses of primary care healthcare utilization and population health outcomes within England (23).

**Figure 2:**
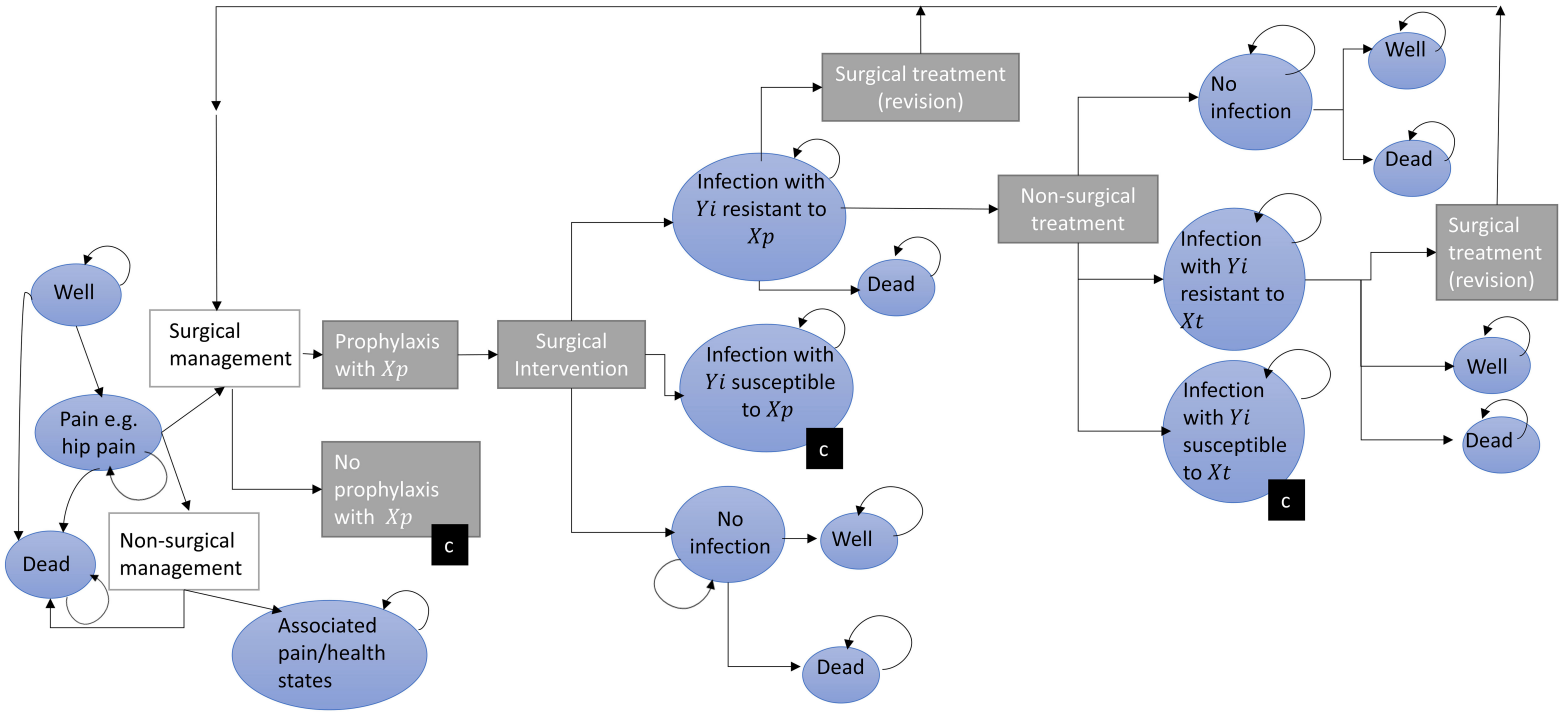
A conceptual model for estimating the impact of antimicrobial resistance on surgery patients. The progress of patients through a surgical management pathway with the potential for infection is depicted following from left to right. Patients initially start (far left) in one of three health states (blue circles) and progress through treatment decisions (rectangles). Circles represent health states, rectangles represent pathway treatment decisions, (c) represents a collapsed branch that mirrors that of another branch within that level (for example the pathway following “No Prophylaxis” includes the same transitions and states as “Prophylaxis.” *Yi* represents microbes where *i* = *1,…,m* different microbes of clinical importance; *Xp,t* represents antibiotics given for prophylaxis (*p* = *1,…,n*) at time *t, Tx* represents antibiotics given for treatment of SSI (*t* = *1,….,q*). It may be that *p* = *t*. The length of time patients spends in each state (indicated by a curved returning arrow) is variable.

Given the median age of elective-surgery patients covered within the national surveillance reports ranges from ~50 to 85 years old (2), long-term care facility data may also be useful, with a Care Quality Commission directory highlighting post-codes of such facilities that can be matched to patient postcodes (24). Additionally, other social care data sources listed in Figure 2 (and described further in Supplementary Table A1) provide information on long and short-term forms of social care that could be useful for costing purposes, if/when applicable to the patient groups of interest.

The cost-of-illness impact, from the NHS (payer and provider) perspective, of surgery, SSIs and drug-resistant infections can be estimated using the aforementioned linked surveillance-administrative datasets to estimate length of stay and/or “Health Resource Group” (HRG) impact (1, 19). If working directly with patient data, the National Costing Grouper, which confers a core Health Resource Group (HRG) to patients' hospital stays, can be used alongside the National Costing Collection workbook (which provides monetary unit costs per HRG) to calculate patient level costs. If working with excess length of stay estimates, the acute patient level activity and costing for 2019–2020 unbundled-activity gives, by speciality within critical care, the total number of days and total cost (£) submitted to the PLICs Acute collection (25), which could hypothetically be used to estimate applicable proxy costs of an excess bed day [e.g., using “Surgical adult patients (unspecified specialty) had a total of 31,807 days and £47,911,153.33 costs across all data submitted”]. For any additional costs of antibiotic prophylaxis or treatments in the hospital or community, unit costs are readily available across the “drugs and pharmaceutical electronic market information tool” (eMIT), the English Prescribing Dataset and the NHS Electronic Drug Tariff for NHS Trust-hospitals, General Practices and community providers, respectively (26).

For AMR and SSI burden estimation from a societal perspective, utilizing standard methods (e.g., human capital methods where each year of life lost is costed to be equivalent to average annual earnings), the use of labor activity and earnings to estimate lost wages through illness and death is needed, and is available (see Supplementary Table A1) (27). However, with these data, those over 60+ and/or 65+ are grouped making it hard to disentangle contributions across cohorts of interest concentrated above 60 years of age, though of course assumptions can be made on the distribution of wage values across ages 60 and 100. Other methods for calculating a value for a statistical life year, which are broader in scope and/or more nuanced in calculation, are available but require more primary data collection in the English context (27).

As well as monetary costs, policy/AMR-scenario impact on population utility, is an important outcome, necessary for cost-utility analyses utilized by national policy makers (28). Such outcomes are generally a function of mortality impacts (as discussed above) and morbidity impacts. Patient-reported outcome measure data, collected by NHS Digital, have been previously linked to other patient data (such as SSISS) to estimate QALY impacts of different infection prevention strategies for primary hip prothesis in England between 2009 and 2012, with more updated data now available (18, 29).

In terms of data access, although all the patient-based data sources outlined in Figure 1 are not fully open-access due to patient identifiable data and subsequent data safeguarding, summary statistics are published openly. Such summary data are often published through annual reports or summary Excel files downloadable from government websites (see Supplementary Table A1), and could be used to estimate incidence rates at a national-level. Access to patient-level data may be permitted subject to asset owner approval processes, allowing for linkage across systems if appropriate data protection protocols are in place.

In the future there may be an increased ease of secondary use of the data sources outlined in Figure 1 for burden estimation purposes through systems similar to Open SAFELY (30). OpenSAFELY was created to allow for urgent research involving electronic health records in primary care (using TPP SystmOne software and EMIS), SGSS, ONS data and A&E attendance data for COVID-19 (30). Researchers write and test code on dummy versions of health data locally, then the code is submitted to be run on secure servers which hold the real versions of the health data, checked in terms of “disclosivity” before then being released for publication purposes (30).

## A conceptual model for estimating the secondary effects for surgery patients

Based on both (i) the existing AMR and SSI literature, and (ii) the data available described above, we propose a state-transition model estimating the primary and secondary effects of AMR, incorporating decision-trees outlining treatment strategies and Markov models outlining potential health states (Figure 2).

For the primary effects, i.e., the direct burden of increased AMR in patients who get surgery and develop SSIs, these pathways can be parameterised with the secondary use of surveillance, administrative and economic data available in England. If outcome data are available at the patient level across hospital stays, statistical models can be utilized to estimate transitions along the patient pathways, adjusting for patient, provider, and socioeconomic characteristics. There are methods available to account for potential sources of bias when dealing with healthcare-associated infections, such as time varying confounding, and other complexities that may occur regardless of study design, such as competing events (19, 21, 31, 32). This allows for more robust patient outcome inclusion, such as cost-of-SSI and mortality impacts (see Supplementary Figure A1 for more detail).

Across the prophylaxis pathway depicted in Figure 2, we highlight that there are the potentially numerous patient pathways with different drug regimens and potential for infection with different organisms. Figure 2 highlights the complexity of this issue, with transition rates and outcomes potentially heterogenous for different drug and bacteria combinations (Supplementary Figure A2 gives a simple example of one drug-bacterial combinations for a portion of this pathway). In practice there is even variation in the antibiotics used across hospitals, with gentamicin and flucloxacillin used for prophylaxis by 57 out of 147 NHS Hospital Trusts and other (differing) antibiotics used across others (33). In a previous cost-effectiveness analysis of strategies to reduce risk of infection following hip replacement therapy, where AMR effects were not incorporated into the equations, a weighted average of the cost of different prophylaxis guidelines between Trusts was used to account for this (18). At an individual level, the specific microbe that caused the infection and type of antimicrobial used to treat it could be important in terms of pathways (and subsequently outcomes and costs), thus the scope of different combinations of prophylactic and therapeutic drug-pathogen exposure definitions (of *Yi* and *Xp,t* in Figure 2 representing different microbes, prophylaxis and treatment antibiotics, respectively) warrant consideration by experts in SSIs depending on the scale of the research question (e.g., local, regional or national).

Inclusion of primary care pathways, in terms of direct effects of AMR on post-surgery patients could be parameterised through linkage of patient data across primary and secondary care settings. Health states presented can have utility values, hospital costs and societal costs attached from Patient Reported Outcome Measure, National Cost Collection and Office for National Statistics datasets, respectively, as discussed in the above section (29, 34, 35). However, expert opinion would be needed to define appropriate case definitions (e.g., time of GP consultation post-surgery/discharge and associated diagnoses “Read” codes). However, for hip surgeries (and other similar, short-stay procedures) it was estimated half of SSIs were captured through readmission surveillance, therefore post-discharge surveillance and linked-HES data to follow (and cost) patients within and across hospitals across the shown post-surgery pathways is key. Average adjusted-wage losses per excess death and days unable-to-work could be estimated through ONS data on employment and wages, and combined with excess deaths and days (e.g., in hospital) counted through (Figure 2) (35).

In a situation of the datasets described in the above section not being accessible at the patient-level, given their coverage they can still be useful in quantifying probabilities of acquiring SSIs. As an example, for hip replacement surgery is under the mandatory surveillance for SSIs, under which 60% undertook continuous surveillance for 2019/20, SSIS reports the incidence of SSI from surveilled hip surgery patients, and the distribution of causative microbes across these SSIs (14). Such reports can therefore be used to estimate transition probabilities across Figure 2 for contracting microbe-specific SSIs following hip replacement surgery in England, alongside assumptions of prophylaxis impact, in the absence of more granular data (see Supplementary Figure A2 for a worked example).

As England has access to longitudinal microbiology data, trends can be determined using these data. Examples of such techniques include simple linear regression or random-walk models, whilst more complex transmission and forecasting modeling methods including seasonality and non-linearity can also be utilized (36). These trends can be included as potential AMR and infection risk scenarios run through the conceptual model (37). However, to parameterise the case of a pan- or extensive-drug-resistant world where current patients would stay in the “no-surgery”/”secondary-effects” pathway, previous cost-effectiveness analyses of the actual surgeries would need to be utilized [for example previous economic evaluations of hip replacement surgeries (38–41)]. There is also the challenge of the increased cost of requiring long-term assisted living for individuals with non-operative management of end-stage osteoarthritis in the elderly population, these costs could be incorporated into Figure 2 by separating out non-surgical management of hip pain and non-surgical treatment of SSIs by settings of care, potentially parameterised by the social care and society demographic data highlighted by Figure 1.

Though previous cost-effectiveness analyses can tell us the general transition probabilities, cost, and utility estimates for a non-surgery scenario, they can't tell us under what AMR and patient-characteristic situations they would occur. Moreover, current SGSS and SSISS data report data on AMR and microbes currently circulating within the healthcare system: a wider scope of scenarios is needed to include microbes and associated drug resistances that may be important in the future, but that aren't currently seen in the data due to low or no numbers (e.g., colistin resistance in Gram-negative infections or a multi-drug resistant *Candida auris*) (6). For this we need expert elicitation of resistance cut-off levels to determine when the “no-surgery scenario” would take effect. Additionally epidemiological forecasting of AMR and infectious disease trends incorporating expert elicitation of predictions for future microbe and AMR importance could be utilized.

## Discussion

We first highlight that there are three potential ways for AMR to impact surgery patients including increasing SSI risk, treatment failure risk and risk of operations being unavailable altogether. Highlighted literature indicates that secondary effects could play a substantial role in the burden of AMR in the future, with estimates for lessening antimicrobial effectiveness including an additional 6,300 deaths per year in the USA and a loss of 2% of world Gross Domestic Product, across different scenarios (4, 42). However, many of the discussed estimates of burden did not sufficiently incorporate uncertainty and/or did not use an explicit mathematical modeling framework that can be practically used and adapted, according to need. A conceptual model utilizing decision trees and Markov models could be used in estimating the potential impacts of AMR on surgery patients, if scoped appropriately and parameterised robustly. The conceptual model constructed within this study highlights the nuance of AMR for SSIs across all pathways, through the acknowledgment of the different potential antimicrobial usage exposures, microbe exposures and treatment options.

The scope for the secondary use of health data in establishing SSI and AMR burden for parameterising transition probabilities, costs and mortality for associated infections occurring in a health system is large. The retrospective use of such data may allow for reduced research burden in parameterising our proposed conceptual model. Even national-level, aggregated data can be used in estimating transition probabilities if data are externally valid. This, in turn, highlights another benefit to SSI surveillance, which already has been shown to reduce SSI rates themselves through benchmarking and outlier identification functions (10). The secondary use of such data, as described here, may be a consideration in the cost-benefit case of public health surveillance itself. Additionally, many of the data sources collated and described within this study are of use in estimating impacts of AMR on other syndromes and clinical specialities, such as respiratory or bloodstream infections treated in primary and secondary care settings.

Earlier published studies have highlighted the need for standardized SSI surveillance protocols such as defined follow up length and data entry methods (12). Our review highlights the benefits of established surveillance systems being able to be readily linked across microbe-, susceptibility- and mortality-surveillance and administrative datasets at the patient-level. Such linkage allows for a greater understanding of the impact of AMR and SSIs on patient outcomes and health system costs, with this being feasible in the NHS through the capture of consistent patient identifiers (unique NHS number, date of birth, sex) across systems (43, 44). However, with patient identifiable and confidential data comes a responsibility to have robust information governance and data protection protocols in place (7, 44). For example, for the surveillance system within England, there is strict adherence to handling patient data in accordance with the Data Protection Act 2018, General Data Protection Regulations (GDPR) and the Caldicott Guidelines (45). Examples of processes that aid this include establishing policy on how long data are held for, who can access the data and how data can be shared. It has been suggested that specifying ethical and privacy principals, and linking these to governance and data access can help with public trust in data capture systems (46), a key factor in having robust data for primary and/or secondary use. With new data access frameworks being explored, such as OpenSAFELY (30), there is increased scope for a reduction in the transfer of patient-level data across parties for research purposes in the future.

However, even with access to current data, data completeness needs to be reviewed. Taking completeness to be in terms of documentation (i.e., are all the available fields filled in and available for use) (47), about 80% of patients in SSISS had a NHS number in a previous analysis, even after doing additional patient tracing to retrieve some missing numbers, and therefore patient-level linkage across numerous datasets may bias subsequent estimates of transitions and outcomes if there are systematic reasons for data non-completeness (18). The completeness of patient data in the SSISS is high for mandatory surveillance in terms of case identification (2), though it is only mandatory to carry out surveillance “for a minimum of 3 consecutive months per financial year in at least one of 4 orthopedic categories: hip replacement, knee replacement, repair of neck of femur or reduction of long bone fracture” (2). This means data for other surgeries may not be fully representative of English surgical patients and SSIs. This could include cesarean or lower bowel surgery patients, who represent a large proportion of the overall burden to the NHS (1, 48). However, the number of operations submitted for 2019/20 for voluntary SSI surveillance showed a 9% increase in comparison to 2018/19, with 27,877 procedures submitted voluntarily in 2019/20 (2). Furthermore, one could use weighting or post-stratification techniques to obtain representative estimates if variables determining selection into the sample are available (49).

Only a few infection types, mainly bacteraemia and notifiable infections, are listed as mandatory surveillance within relevant data capture systems, and as such the epidemiological data for other pathogens could be biased. However, such data has been routinely used to present AMR data at the national level in England (14) and a 2020 report comparing mandatory and voluntary submissions found a high ascertainment rate comparing across the systems (for bacteria present in both systems) (50). In the absence of surveillance systems, routinely collected HES data may be useful for infection rates and patient outcomes, with routinely collected data to estimate rates of SSIs being found to have sensitivities ranging from 60 to 98% (10, 51), though this would likely not provide information on microbe or AMR.

Though PROMs data are theoretically available for certain patients, it is only available for certain surgeries (hip and knee replacement) (52). Moreover, even when these data are available, they may not be useful for our intended purpose. A previous analysis had to revert to using literature as PROM's data weren't available for their SSI case definitions (e.g., within 14 days of the date of infection) (18). For international comparisons where Disability-Adjusted Life Years may be wanted (instead of Quality-adjusted life years), a large European study is available, where disability weights for SSI states are based on previous observational studies which have elicited utility values (53). From a patient perspective, administrative datasets in NHS England described in this review do not account for patient-level costs, though these data may be available in insurance-based healthcare systems (7).

Regarding the HRG and excess length of stay unit costing, England is currently undergoing the NHS England and NHS Improvement's Costing Transformation Programme. This was piloted in 2016 and had annual stages of increased implementation subsequently. Therefore, currently such data come with potential data quality issues given these are the first few years of the new Patient-Level Information and Costing system, though this bias should decrease over time if systems and processes remain unchanged (54).

Even with complete and secure data systems in place, this review deduces that the secondary use of health data cannot be used solely to parameterise a secondary effects model for AMR and SSIs. Prospective trials of SSI prevention measures, patient and public elicitation for utility values, alongside expert elicitation studies for “post-antibiotic” scenario understanding are needed. A 2019 literature review calls for more evidence from primary studies on the intervention effectiveness of different SSI prevention techniques (12). Such data normally come from randomized-control trials rather than secondary use of health data, although even trials are now making use of routinely collected data to inform primary trials and/or for longer follow-up period (55).

Additionally, the model currently depicts a simplified picture of “prophylaxis” vs. “no prophylaxis” comparison of surgical management pathways, as in addition to antimicrobial prophylaxis, there are several interventions to prevent SSIs, such as using sterile gowns or changing surgical instruments prior to wound closure (12), many of which are recommended for surgery undertaken in England (56). These pathways can be added to the core framework outlined here as and when necessary, with scope of the model pathways best extended based on expert opinion for specific surgeries or settings. As has been done in more general AMR burden estimation models (37). The proposed conceptual model recommends AMR scenario dynamics be included through external trend analyses and expert elicitation to then feed epidemiological parameters directly into the state transition model, which has been done in more general AMR burden estimation models (37). The conceptual model could be expanded into incorporate transmission dynamics but would require more health states (representing other reservoirs of antibiotic usage and resistance) and therefore potentially more data. The model can also be run for different intervention scenarios related to antimicrobial stewardship and/or SSIs, subsequently comparing costs and effects across scenarios to determine the cost-effectiveness of such interventions.

While here we do not make specific recommendations related to the general health economic approach of quantifying our conceptual model, general guidelines are available elsewhere for health economic modeling (28, 57), the reporting of which is currently lacking from some studies that have attempted to quantify secondary effects of AMR (4, 42). Based on standard guidelines, the England case study should take an NHS perspective [as recommended by NICE for the base case (28)], cover the lifetime of a hypothetical cohort to capture the potential long-term impacts, and use a 3.5% discount rate for future costs and a 1.5% discount rate for quality-adjusted life years declining over 30 years as recommended by the Treasury (58). Using this approach, parameter and methodological uncertainty can be tested by varying parameter values, discount rates and time horizon through one-way and probabilistic sensitivity analyses. Structural uncertainty and heterogeneity could also be explored in further iterations of Figure 1, by adapting pathways and including specific subgroups if sufficient data are available. With such modeling approaches, a broader perspective is enabled through the inclusion of labor productivity cost proxied by national wage and employment data (59). However, there may be a need in the future to explore the effects of presenteeism impacts (i.e., incorporate not only loss of work productivity through hospital stay or death, but also general loss of work productivity for patients along different pathways) (60), and also explore informal market production impacts (27, 59).

Though we have currently focused on the secondary use of health data in the NHS, the findings are applicable to settings where similar datasets are available. A 2018 review found 56 healthcare-associated infection and AMR surveillance systems from 20 countries within Europe, with 32 SSI surveillance systems included (61). This indicates that there is already a large potential resource for understanding the secondary effects of AMR considering the proposal outlined in our review. We have also focused on SSIs, but secondary use of cancer patient data may be explored in a similar manner; through national cancer registration data linked to other surveillance and administrative data (44).

In conclusion, AMR is a complex phenomenon which has the potential to alter health outcomes for patients who contract drug resistant SSIs and change surgery patient pathways due to secondary effects. Though the secondary use of health data, in the English setting, has the potential to parameterise models quantifying the former, it falls short of being able to quantify the latter in isolation. However, such data can be combined with expert elicitation to parameterise a health state transition model that incorporates primary and secondary impacts of AMR on surgery patients over time. With growing SSI and AMR surveillance systems globally, alongside expert elicitation and investigations into potential future epidemiological scenarios, we can begin to understand the potential secondary effects of AMR through the application of the proposed conceptual model in other settings, and therefore understand how to deal with this phenomenon more efficiently.

## Author contributions

NN and SE reviewed the literature, reviewed the datasets, constructed the conceptual model diagram, and wrote the initial drafts of the manuscript. JR managed the project and contributed to study design. JR and RA secured funding for the project. KP, RT, TL, BM-P, and GK provided technical guidance on data and methods and/or literature cited. All authors aided in drafting the manuscript and approved the submitted manuscript.

## Funding

The research was funded by the National Institute for Health Research Health Protection Research Units (NIHR HPRU) in Healthcare Associated Infections and Antimicrobial Resistance at Imperial College London (grant number HPRU-2012–10047 funding NN's time and with NN subsequently holding an honorary contract through HPRU-200876) and the University of Oxford (grant number HPRU-2012-10041), all in partnership with Public Health England (PHE) [now United Kingdom Health Security Agency (UKHSA)]. GK was supported by a fellowship from the UK Medical Research Council (MR/P014658/1).

## Conflict of interest

The authors declare that the research was conducted in the absence of any commercial or financial relationships that could be construed as a potential conflict of interest.

## Publisher's note

All claims expressed in this article are solely those of the authors and do not necessarily represent those of their affiliated organizations, or those of the publisher, the editors and the reviewers. Any product that may be evaluated in this article, or claim that may be made by its manufacturer, is not guaranteed or endorsed by the publisher.

## Author disclaimer

The views expressed are those of the author(s) and not necessarily those of the NHS, the NIHR, the Department of Health, or the United Kingdom Health Security Agency (formerly Public Health England).

## Abbreviations

AMR: antimicrobial resistance
HES: hospital episode statistics
NHS: national health service
ONS: office for national statistics
PROMs: patient reported outcome measures
SGSS: second generation surveillance system
SSI: surgical site infection
SSISS: SSI surveillance service.
