# Supplementary material for: Quantifying the primary and secondary effects of antimicrobial resistance on surgery patients: Methods and data sources for empirical estimation in England
Source: Front Public Health. 2022 Aug 8;10:803943. doi: 10.3389/fpubh.2022.803943 (PMC9413182; doi:10.3389/fpubh.2022.803943)
Supplement: Supplementary file 1 [file Data_Sheet_1.docx]

Supplementary Material

# Table A1. Summary of Health and Economic Data Sources Discussed for Antimicrobial Resistance Burden Estimation for Surgery Patients in England

Datasets are listed in alphabetical order where possible. *The list of variables provided in this column may not be exclusive but describes the types of data held relevant to this study. ^Ϯ^ Datasets listed as ‘United Kingdom Health Security Agency’ were managed by Public Health England at the time of reference sourcing but are transferred to the United Kingdom Health Security Agency following October 1^st^, 2021.

| Dataset  *[Information asset manager]* | Primary Purpose | Data Available* | References |
| --- | --- | --- | --- |
| Adult Social Care Finance Return (ASC-FR) & Short and Long Term Services (SALT) collection  [*NHS Digital*] | The Adult Social Care Activity and Finance data files are produced from a combination of the ASC-FR and SALT collections, collected from Councils with Adult Social Services Responsibilities (CASSRs) in England to provide insight into adult social care activity and expenditure on an annual basis. | This dataset contains by region, age bands and care type (long term versus short term, or specific support type such as “physical support” provided in a residential home) data on; expenditure (gross and net), income (total and by source), unit costs and number of patient/client completed episodes, number of clients accessing care and new requests for care. | (1) |
| Analysis of population estimates tool  *[Office for National Statistics (ONS)]* | The aim of the ONS is to ‘collect, analyse and disseminate statistics about the UK's economy, society and population.’ All the cited datasets from ONS are stated to ‘have a wide range of uses’ by ‘Central government, local government and the health sector use them for planning, resource allocation and managing the economy.’ | This dataset provides an ‘interactive analysis of estimated annual, mid-year population changes for England and Wales, by geography, age and sex.’ | (2, 3) |
| Annual Survey of Hours and Earnings (ASHE)  *[Office for National Statistics (ONS)]* | The aim of the ONS is to ‘collect, analyse and disseminate statistics about the UK's economy, society and population.’ All the cited datasets from ONS are stated to ‘have a wide range of uses’ by ‘Central government, local government and the health sector use them for planning, resource allocation and managing the economy.’ | The Annual Survey of Hours and Earnings contains estimates of earnings for employees, including (but not limited to):   - gross weekly pay - weekly pay excluding overtime - overtime pay - gross hourly pay - gross annual pay   These data are available by sex and full-time or part-time status, by region, occupation, industry, age group and public or private sector. | (4) |
| Clinical Practice Research Datalink (CPRD)  (~700 GP Practices)  [*Department of Health and Social Care*]  AND  The Health Improvement Network (THIN)  (~600 GP Practices)  [*The Health Improvement Network Ltd*]  AND  QResearch  (~1500 GP Practices)  [*University of Oxford and EMIS*]  AND  ResearchOne  (~400 GP Practices)  [*The Phoenix*  *Partnership (TPP) and the University of Leeds*] | Each of these data sources are based on information from GP practice administrative, which use hierarchical clinical coding systems. The primary purposes of the administrative electronic health record systems have been previously stated to mainly include clinical practice (aiding in the recall of previous treatment or diagnoses to clinicians). However other functions are financial (billing and budgeting) and statistical (such as research use). | Data variables are those based on the result of GP consultations. This includes patient characteristics (NHS number, birth month and year, marital status etc.), practice characteristics (unique identifier, region), consultation characteristics (staff, dates, type, medical codes), test and treatment characteristics (product/test types, amounts, results (for tests), dosages (for treatments).  *Please note that much information here is taken from the cited Kontopantelis 2017 paper and as such exact numbers and descriptors may have changed subsequently.* | (5-9) (10) |
| CQC Locations  [*Care Quality Commission (CQC)*] | A directory of providers that offer care that are registered with the CQC, whereby the CQC monitor, inspect and regulate services offered (including for care homes, dentists, hospitals, General Practitioner Practices, community and mental health services). | The weekly-updated dataset, provides the following variables for each registered facility; Name, Also known as, Address, Postcode, Phone number, Service’s website (if available), Service types, Date of latest check, Specialisms/service, Provider name, Local Authority, Region, Location URL, CQC Location, CQC Provider ID. | (11, 12) |
| Deaths registered weekly/monthly in England and Wales  *[Office for National Statistics]* | Surveillance of mortality events based on all death registrations in England. | Total counts of death by sex, age and week or month are available through this dataset. Non-open-access data extracts also include patient level data such as NHS number, date of birth, date of death and listed cause of death. | (13-15) |
| Drugs and pharmaceutical electronic market information tool (eMIT)  [*Department of Health and Social Care*] | To provide information about the prices and usage for generic drugs/pharmaceutical products used by NHS hospitals. | The data is based on “Pharmex” data (data on 13 million line-order-entries for over 10,000 products), held by the Commercial Medicines Unit. The data eMIT provides (for the specified 12month period) includes:  (i) Drug name and pack-size.  (ii) Quantity: an estimate of NHS hospital-sector usage from English trusts for each of the products.  (iii) Weighted average price: the average price paid for that product over the last 4 months of the period.  (iv) A measure of how much that average changed. | (16) |
| English Prescribing Dataset (EPD)  [*NHS Business Services Authority*] | This dataset aims to collect and provide community-level prescribing information to NHS stakeholders primarily to aid performance management, financial planning and improve clinical practice. | It includes for each General Practitioner (GP) Practice (at the GP-level)/cost-centre: GP identifiers, prescribed and dispensed medicines and dressings (by British National Formulatory Chapter, Chemical Substance and Presentation); prescribed and dispensed dressings and appliances; total number of items, the quantity for each individual item, the total quantity prescribed and dispensed, the ‘Net Ingredient Cost’ ) (based on published prices), the ‘Actual Cost’ (accounts for the national average discount and some payments to dispensers), average Daily Quantity (the typical daily dose of a medication, prescribed to adult patients. | (17, 18) |
| Estates Returns Information Collection (ERIC)  *[NHS Digital]* | To serve as a mandatory collection for all NHS organisations providing NHS funded secondary care, in England, to monitor ‘efficiencies and funding of the NHS estate’. | Trust code, name and type (e.g. acute teaching versus community) data are available. Additionally, it houses a breakdown of costs of providing, maintaining and/or consuming the NHS Estate including buildings, equipment, utilities and services such as food and laundry. | (19) |
| Hospital Episode Statistics (HES)  *[NHS Digital]* | Its primary purpose is to facilitate the payment for services from NHS England to NHS hospitals and independent sector health care providers (providing NHS-commissioned care). | Data available include; Trust code, patient characteristics (such as NHS number, age and sex, postcode), episode characteristics (such as admission and discharge dates) and procedures undertaken (such as type of surgery) during hospital admission, recorded outpatient service appointments and attendance at Accident & Emergency units. | (20-22) |
| IQVIA Hospital Treatment Insights Service  [*IQVIA*] | This dataset is used to describe and understand ‘how drugs are being used and to conduct studies to monitor the use, effectiveness and safety of drugs when treating diseases’ within the hospital setting. | This dataset contains HES fields and antibiotic prescription information (in non-patient-identifiable form the NHS number has been removed). Information collected by NHS Trusts includes “a patient’s Name, Address, Post Code, NHS Number and Date of Birth, only the hospital Trust and NHS Digital hold these details. NHS Digital links the data to its Hospital Episode Statistics database, then removes all personal details before the data is sent to IQVIA.” | (23) |
| Labour Force Survey (LFS)  *[ONS]* | The aim of the ONS is to ‘collect, analyse and disseminate statistics about the UK's economy, society and population.’ All the cited datasets from ONS are stated to ‘have a wide range of uses’ by ‘Central government, local government and the health sector use them for planning, resource allocation and managing the economy.’ | There are different individual datasets available based on the LFS available through the ONS website, such as “A05 SA: Employment, unemployment and economic inactivity by age group (seasonally adjusted)”, which provides employment, unemployment, activity and inactivity rates by sex and age groups. | (24) |
| National Cost Collection  *[NHS Digital, NHS England & NHS Improvement]* | To provide insights on the cost of care and how money is spent across the NHS to then help support delivery of ‘high-quality care’ and ‘better value’, nationally mandated across all secondary and tertiary care providers.  The National Cost Collection (NCC) is based on the Patient Level Information Costing System data set, which is used to; inform new methods and approaches of pricing NHS services, inform the association of costs of care with provider characteristics and patient characteristics, and inform benchmarking for regulatory purposes. | Previously, NHS Reference cost reports were published within annual reports based on aggregated cost data submitted by NHS Trusts (such as unit cost per excess bed day). However, as part of the Costing Transformation Programme, National Cost Collection patient stay average costs are now estimated.  Internal Trust-based Patient Level Information and Costing Systems will have patient characteristics, care characteristics and cost and income per patient per hospital episode estimates. The National Costing Grouper confers a core Health Resource Group (HRG) to every finished consultant episode of care for a patient. With access to the patient-level data one could use the NCC workbook, published HRG unit costs and the HRG grouper to calculate patient level costs (see referenced NHS Digital websites for more detail on this) as done at the Trust-level. The National Tariff annex provides HRG Name, "Outpatient procedure tariff (£)", "Combined day case /ordinary elective spell tariff (£)","Day case spell tariff (£)","Ordinary elective spell tariff (£)","Ordinary elective long stay trim point (days)","Non-elective spell tariff (£)","Non-elective long stay trim point (days)","Per day long stay payment (for days exceeding trim point) (£)" and information on Reduced short stay emergency tariffs applicable to that HRG.  Additionally, total number and cost of crucial care days are submitted to the Patient Level Information and Costing System Acute collection, by critical care unit function (such as “non-specific general, adult critical care patients predominate” and “Surgical adult patients (unspecified speciality)”. The acute patient level activity and costing for 2019-2020 (unbundled activity) has been published by total and trust provider level. This gives, by speciality within critical care, the total number of days and total cost (£) submitted to the PLICs Acute collection. | (25-27) |
| NHS Electronic Drug Tariff  [*NHS Business Services Authority*] | The Drug Tariff, which is sent to Pharmacies and GP Practices, outlines what will be paid to pharmacy contractors for NHS services provided either for reimbursement or for remuneration and rules to be observed when providing such services. | The monthly produced online and offline data can be used to estimate the basic cost price of drugs used and supply cost in community settings through:   - The basic prices of the drugs (and appropriate deductions as stated in the tariff). This includes drug, quantity of drugs, basic price and availability of generic versions of the drug. - Professional fees. - Cost of consumables and containers as set out in the tariff. | (28) |
| National life tables: UK  *[ONS]* | The aim of the ONS is to ‘collect, analyse and disseminate statistics about the UK's economy, society and population.’ All the cited datasets from ONS are stated to ‘have a wide range of uses’ by ‘Central government, local government and the health sector use them for planning, resource allocation and managing the economy.’ | This data can be used to estimate background mortality rates, as this provides (i) the central rate of mortality, (ii) the mortality rate between age x and (x +1), that is the probability that a person aged x exact will die before reaching age (x +1), (iii) the number of survivors to exact age x of 100,000 live births of the same sex who are assumed to be subject throughout their lives to the mortality rates experienced in the three year period to which the National Life Table relates, (iv) the number dying between exact age x and (x +1) and (v) the average number of years that those aged x exact will live thereafter. | (29) |
| National Joint Registry (NJR)  *[Healthcare Quality Improvement Partnership (HQIP)]* | The purpose of the NJR data collection is to provide an early warning system for patient safety issues.  Furthermore, ‘to continue to improve quality and cost-effectiveness of care, data collected about joint replacement surgery in the UK are used to report on, and monitor, patient outcomes and to support research.’ | From this registry, data on the types of procedures performed (including surgical and incision approach) and if/when a revision was performed on the patient for hip, knee, ankle, elbow and shoulder joint replacements. The full non-open-access dataset includes patient identifiers (including NHS number and date of birth). | (30) |
| The national SSI Surveillance Service (SSISS)  *[United Kingdom Health Security Agency ^Ϯ^]* | Active surveillance programme collecting data on surgery and related SSIs submitted by hospitals through prospective follow-up of patients. Data are used to enhance the quality of patient care by providing national benchmarks which local providers can compare themselves to. Seventeen categories of major surgery are included within the SSISS, with surveillance targeting open surgical procedures, with orthopaedic surgery SSI surveillance mandatory. | Patient-level and surgery-related characteristics by surgical category, SSI characteristics (such as incidence and time to infection, inpatient and/or readmission, causative organism), patient and surgical risk factors. The full non-open-access dataset includes patient identifiers (including NHS number and date of birth). | (13, 31, 32) |
| Patient Reported Outcome Measures (PROMs)  *[NHS Digital]* | PROMs are a means of collecting information on the effectiveness of care delivered to NHS patients as perceived by the patients themselves. | The patient- and episode-level dataset provides patient answers to (i) condition specific measures for self-reported health status (such as the Oxford Hip Score (OHS) questions), (ii) generic measures for self-reported health status (such as the EQ-5D) and additional questions about patients’ general health. Additionally, the dataset has patient-identifiable information for linkage (not available for general, wider analyses). This is available for hip and knee replacement surgery patients (those that complete it), it was historically also available for groin hernia and varicose vein surgeries. | (13, 33) |
| The Healthcare Associated  Infection Data Capture System (HCAIDCS) and The Second-Generation Surveillance System (SGSS)  *[United Kingdom Health Security Agency ^Ϯ^]* | The system captures results from NHS laboratories to provide early detection and longer-term trends in changes in epidemiology, enabling the protection of public health.  The system aims to determine burden of infectious disease (including epidemiology and mortality), enable timely action if necessary, monitor intervention impact, provide information to support the development of guidance on the clinical management of patients, inform material provided to the general public about infectious disease risks and ensure that the UK makes its full contribution to international efforts to protect health.  The Second Generation Surveillance System (SGSS) is a voluntary surveillance data, whilst the mandatory surveillance data reported to PHE via a real-time web-based surveillance system (Healthcare Associated  Infection Data Capture System (HCAIDCS)). | Data available include patient characteristics (such as NHS number, age and sex), organism characteristics (type of microbe and antibiotic susceptibility - in some cases), specimen characteristics (date and sample-type taken, source and reporting laboratories).  Reporting and publication of data openly is on specific microbes that are determined to be of particular interest by the government advisory group, requiring mandatory reporting from NHS trusts (this includes *Methicillin-resistant Staphylococcus aureus* bacteraemia*, Methicillin-sensitive Staphylococcus aureus* bacteraemia*, Escherichia coli* bacteraemia*, Klebsiella spp.* bacteraemia, *Pseudomonas aeruginosa* bacteraemia and *Clostridium difficile* infections). | (34-36) |

# Figure A1. Potential Statistical Modelling Processes for Surgery Treatment Pathways within Hospitals

T0 is the individual-level start time (when surgery occurs) whereby outcomes of interest are observed before T2 (study end date). A time-dependent data set (with daily time intervals) can be defined including variables to highlight; T0, time of infection (T1), time of discharge and/or death. Multi-state model processes may be split into inverse probability weighting and/or cox-proportional hazards models to model the risk of experiencing infection events, discharge events and death events across either and/or both processes 1 and 2. Potential confounding impacts of different prophylaxis, treatment protocols, patient and hospital characteristics can be integrated into these models as seen in previous literature (37-39).


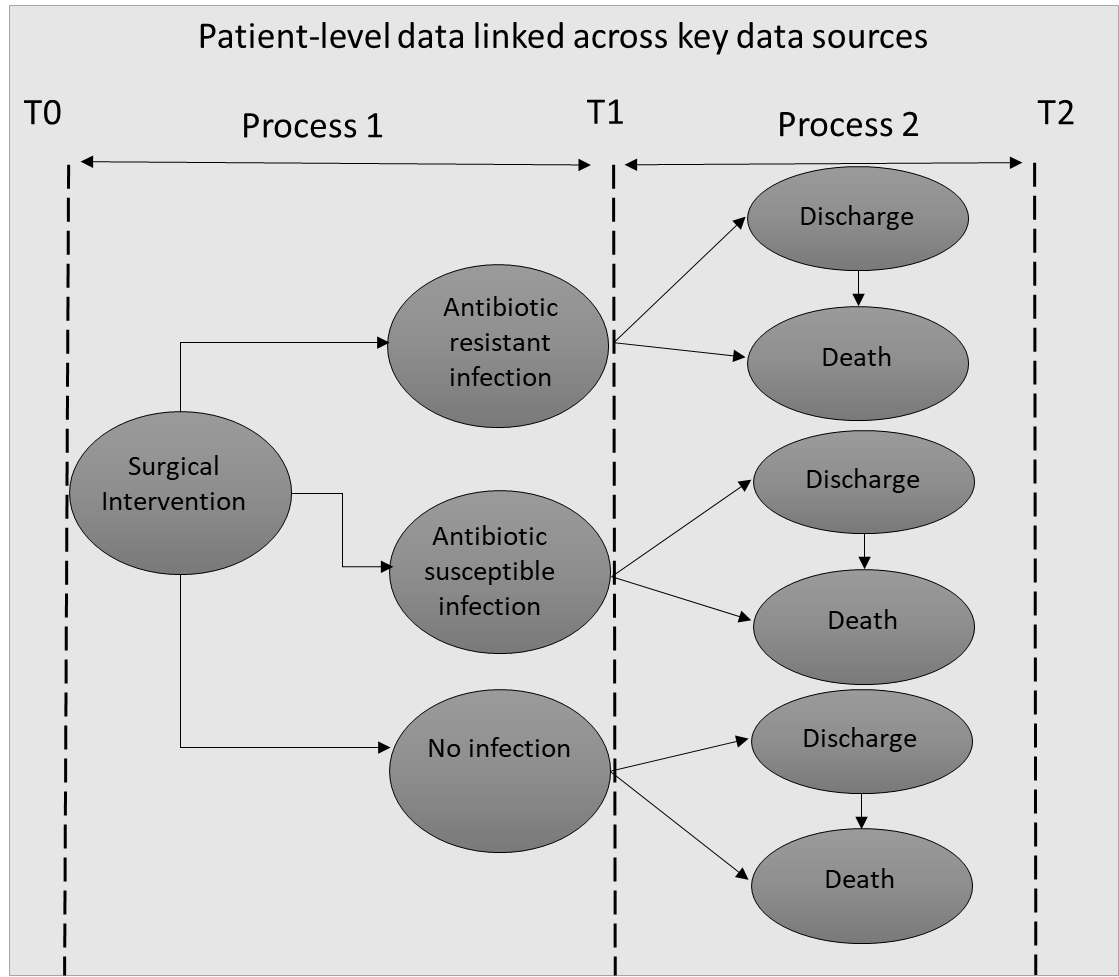


# Figure A2. Example Calculation of Initial Base Case Transitions for Staphylococcus aureus Surgical Site Infections using Surveillance Data

All numerical values presented are taken from the 2018-19 SSISS report (40), focusing on inpatients with monomicrobial methicillin resistant and/or methicillin susceptible surgical site infections between April 2014 to March 2019. [c] represents a collapsed branch that mirrors that of another branch within that level. *This would need adjusting under assumptions about prophylaxis efficacy against *S. aureus* and how many of the SSIs within the data come from the no prophylaxis vs prophylaxis group.


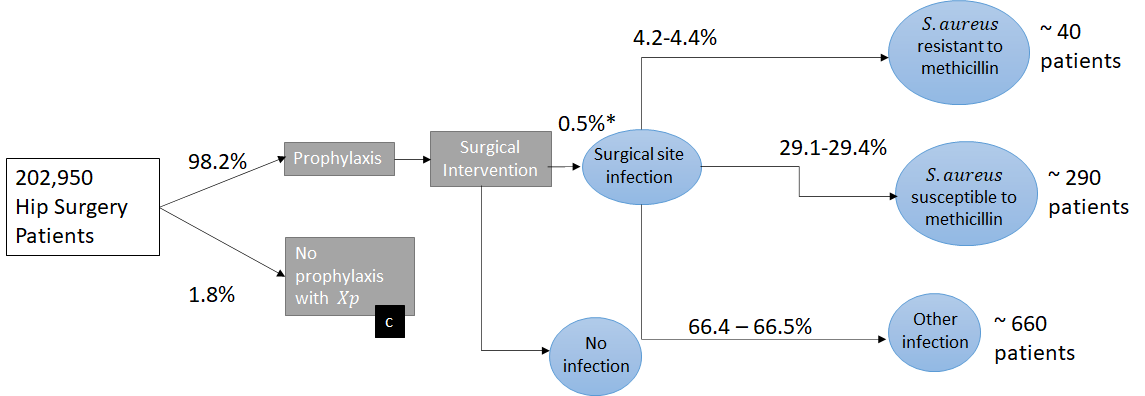


# References

1. NHS Digital. Adult Social Care Activity and Finance Report, England - 2019-20. 2020 [Available from: <https://digital.nhs.uk/data-and-information/publications/statistical/adult-social-care-activity-and-finance-report/2019-20#resources>.

2. Office for National Statistics. Population and migration [Available from: <https://www.ons.gov.uk/peoplepopulationandcommunity/populationandmigration>.

3. Office for National Statistics. Analysis of population estimates tool [Available from: <https://www.ons.gov.uk/peoplepopulationandcommunity/populationandmigration/populationestimates/datasets/analysisofpopulationestimatestool>.

4. Office for National Statistics. Annual Survey of Hours and Earnings (ASHE) [

5. Chaudhry Z, Mannan F, Gibson-White A, Syed U, Ahmed S, Kousoulis A, et al. Outputs and growth of primary care databases in the United Kingdom: bibliometric analysis. Journal of innovation in health informatics. 2017;24(3).

6. Medicines & Healthcare Products Regulatory Agency & National Insitute for Health Research. Clinical Practice Research Datalink. 2021 [Available from: <https://www.cprd.com/>.

7. The Health Improvement Network Ltd. About us 2021 [Available from: <https://www.the-health-improvement-network.com/about>.

8. QResearch. About ​​​QResearch. 2021 [Available from: <https://www.qresearch.org/about/>.

9. Kneale D, Khatwa M, Thomas J, (EPPI-Centre UIoE. Identifying and appraising promising sources of UK clinical, health and social care data for use by NICE. EPPI-Centre, Social Science Research Unit.: UCL Institute of Education, University College London, London.; 2016.

10. McMillan B, Eastham R, Brown B, Fitton R, Dickinson D. Primary Care Patient Records in the United Kingdom: Past, Present, and Future Research Priorities. J Med Internet Res. 2018;20(12):e11293.

11. Care Quality Commission. What we do. 2021 [Available from: <https://www.cqc.org.uk/what-we-do>.

12. Care Quality Commission. CQC care directory - zip [updated 29/09/2021. Available from: <https://www.cqc.org.uk/files/cqc-care-directory-zip>.

13. Graves N, Wloch C, Wilson J, Barnett A, Sutton A, Cooper N, et al. A cost-effectiveness modelling study of strategies to reduce risk of infection following primary hip replacement based on a systematic review. Health Technol Assess. 2016;20(54):1-144.

14. Health and Social Care Information Centre. A Guide to Linked Mortality Data from Hospital Episode Statistics and the Office for National Statistics. 2015.

15. Office for National Statistics. Deaths registered weekly in England and Wales, provisional 2021 [Available from: <https://www.ons.gov.uk/peoplepopulationandcommunity/birthsdeathsandmarriages/deaths/datasets/weeklyprovisionalfiguresondeathsregisteredinenglandandwales>.

16. UK Department of Health and Social Care. Drugs and pharmaceutical electronic market information tool (eMIT) 2011 [Available from: <https://www.gov.uk/government/publications/drugs-and-pharmaceutical-electronic-market-information-emit>.

17. NHS Business Services Authority. Prescription data 2021 [Available from: <https://www.nhsbsa.nhs.uk/prescription-data>.

18. NHS Business Services Authority. English Prescribing Dataset (EPD) 2021 [

19. NHS Digital. Estates Return Information Collection (ERIC) 2018/19: Data Quality Report 2019. 2019.

20. Digital N. Hospital Episode Statistics (HES) Analysis Guide. December 20192019.

21. Team HDQ. The HES Processing Cycle and HES Data Quality. In: Digital N, editor. 2016.

22. Boyd A, Cornish R, Johnson L, Simmonds S, Syddall H, Westbury L, et al. Understanding Hospital Episode Statistics (HES). London, UK: CLOSER. 2017.

23. IQVIA. Hospital Treatment Insights Service 2021 [Available from: <https://www.iqvia.com/locations/united-kingdom/information-for-members-of-the-public/hospital-treatment-insights-service>.

24. Office for National Statistics. A05 SA: Employment, unemployment and economic inactivity by age group (seasonally adjusted) [Available from: <https://www.ons.gov.uk/employmentandlabourmarket/peopleinwork/employmentandemployeetypes/datasets/employmentunemploymentandeconomicinactivitybyagegroupseasonallyadjusteda05sa>.

25. NHS England and NHS Improvement. 2020/21 National Tariff Payment System. 2020.

26. NHS England and NHS Improvement. Volume 4: National Cost Collection – community sector. 2020.

27. NHS England and NHS Improvement. Volume 6: National Cost Collection – community sector. 2020.

28. NHS Business Services Authority. NHS Electronic Drug Tariff 2021 [Available from: <https://www.drugtariff.nhsbsa.nhs.uk/#/00805984-DC/DC00805981/Home>.

29. Office for National Statistics. National life tables – life expectancy in the UK: 2017 to 2019 2020 [Available from: <https://www.ons.gov.uk/peoplepopulationandcommunity/birthsdeathsandmarriages/lifeexpectancies/bulletins/nationallifetablesunitedkingdom/2017to2019>.

30. National Joint Registry. NJR Reports [Available from: <https://reports.njrcentre.org.uk/>.

31. Troughton R, Birgand G, Johnson AP, Naylor N, Gharbi M, Aylin P, et al. Mapping national surveillance of surgical site infections in England: needs and priorities. J Hosp Infect. 2018;100(4):378-85.

32. Public Health England. Surveillance of surgical site infections in NHS hospitals in England: April 2019 to March 2020. 2020.

33. Secondary Care Analysis (PROMs) NHS Digital. Patient Reported Outcome Measures (PROMs) in England. 2017.

34. Public Health England. AMR local indicators 2021 [Available from: <https://fingertips.phe.org.uk/profile/amr-local-indicators/data>.

35. Public Health England. English Surveillance Programme for Antimicrobial Utilisation and Resistance (ESPAUR) Report 2019 to 2020. 2020.

36. Public Health England. Mandatory Healthcare Associated Infection Surveillance: data quality statement: October 2020. 2020.

37. Pouwels KB, Vansteelandt S, Batra R, Edgeworth J, Wordsworth S, Robotham JV, et al. Estimating the Effect of Healthcare-Associated Infections on Excess Length of Hospital Stay Using Inverse Probability-Weighted Survival Curves. Clin Infect Dis. 2020;71(9):e415-e20.

38. Pouwels KB, Van Kleef E, Vansteelandt S, Batra R, Edgeworth JD, Smieszek T, et al. Does appropriate empiric antibiotic therapy modify intensive care unit-acquired Enterobacteriaceae bacteraemia mortality and discharge? J Hosp Infect. 2017;96(1):23-8.

39. Coeurjolly JF, Nguile-Makao M, Timsit JF, Liquet B. Attributable risk estimation for adjusted disability multistate models: application to nosocomial infections. Biom J. 2012;54(5):600-16.

40. Public Health England. Surveillance of surgical site infections in NHS hospitals in England: April 2018 to March 2019. 2019.
